# Innovation in Times of Crisis: How Civil Protection Organizations in Europe Coped and Adapted During the COVID-19 Pandemic

**DOI:** 10.1007/s41125-023-00090-6

**Published:** 2023-05-09

**Authors:** Florian Roth, Benjamin Kaluza, Katharina Pfeffer, Esther Rümelin, Joel Kirchner, Maike Overmeyer, Florian Neisser, Thomas Jackwerth-Rice, Aleyna Kilicaslan, Johannes Sautter

**Affiliations:** 1 Zurich University of Applied Sciences, Winterthur, Switzerland; 2 Fraunhofer Institute for Technological Trend Analysis INT, Euskirchen, Germany; 3 Fraunhofer Institute for Systems and Innovation Research ISI, Karlsruhe, Germany; 4 Fraunhofer-Institute for Industrial Engineering IAO, Nobelstr. 12, 70569 Stuttgart, Germany

**Keywords:** COVID-19, Pandemic, Civil protection, Organizations, Resilience, Innovation

## Abstract

This explorative study provides insights into how civil protection organizations can learn, be innovative, and develop their capabilities toward achieving greater organizational resilience. After the enormous impact of the COVID-19 pandemic, civil protection organizations had to actively shape crisis-related learning processes, particularly concerning workflows and organizational structures, collaboration with stakeholders, preparedness, risk analysis, digitalization, and the political framework. Empirical findings collected in Europe during and between the main pandemic peaks of 2020 and 2021 were analyzed for implications for policymakers and civil protection organizations. Key findings illustrate how important it is for civil protection organizations to respond quickly and adapt flexibly in times of crisis. In interviews with civil protection experts, respondents highlight the benefits of a partner network and the need for digital capabilities and data infrastructure. Other relevant factors in dealing with a crisis were found to be self-evaluation and the ability to recover from a previous wave while preparing for and responding to the next.

**Supplementary Information:**

The online version contains supplementary material available at 10.1007/s41125-023-00090-6.

## Introduction

Recent global challenges such as climate change, mass migration, and especially the COVID-19 pandemic, demonstrate the importance of strengthening systemic resilience (Glavovic & Smith 2014; Kelman et al. 2016; Walker 2019; Ansell et al. 2021). Civil protection organizations play a key role in crisis preparedness, response, and recovery. Using the example of the COVID-19 pandemic, this explorative study examines the problems faced by civil protection organizations in dealing with complex and protracted crises and identifies the capabilities they develop that can strengthen their resilience. Special focus was placed on the resources that enable organizations to learn valuable lessons and develop innovative approaches for the future (Teo et al. 2017; Schomaker & Bauer 2020).

The pandemic that hit Europe in early 2020 after the SARS-CoV-2 outbreak and put unprecedented pressure on civil protection organizations all over the world is an opportunity to document and analyze measures taken and identify ways to deal with a protracted crisis of such magnitude in future. It enabled us to explore the conditions under which organizational resilience evolves, how an organization's crisis management can be enhanced, and how capabilities can be developed and actively fostered. Our empirical analysis drew on interviews with representatives of civil protection agencies and independent experts from five European countries. This survey was conducted between June and September 2021 as part of a larger research project on how the COVID-19 pandemic affected and was handled by governments, private sector enterprises, and society at large (Mueller & Sautter 2022).

As our findings confirmed, the COVID-19 crisis acted as an innovation driver for the civil protection organizations we studied, resulting in new organizational structures, workflows, and tools. The organizations learned to improvise and adapt in order to cope with the new demands, often by drawing on preexisting, informal networks and trusting the expertise of their staff to find context-sensitive solutions.

Among other things, we wanted to see whether the established three-staged cyclic model of crisis management (anticipation, coping, adaptation) is applicable for the coping with pandemics in pandemics (Duchek 2020). Findings suggest the model may be ill-suited to capture these dynamic learning and innovation processes, not least because during the COVID-19 pandemic, preparedness measures, acute response efforts, and adaptive steps often ran in parallel. We also identified barriers to adaptation and innovation processes in civil protection organizations. On the other hand, we found several levers for increasing the resilience of such players. These include, among others, bold investments in digital capabilities, the establishment of systematic knowledge management and learning processes, and a clear division of labor between civil protection experts and politicians.

The paper is structured as follows: Chapter 2 gives an overview of the challenges of civil protection organizations in times of crisis, focusing on capabilities fostering organizations’ learning and innovation processes under the extreme conditions of a global health crisis. Chapter 3 elaborates on the conceptual framework of the empirical analysis, the design of the interview study, and the composition of the expert pool. In Chapter 4, the results of the study are clustered into five thematic areas that appear to be particularly significant for crisis management and the organizational resilience capabilities needed for this purpose: (a) workflows and organizational structures, (b) internal and external collaboration, (c) planning and risk analysis, (d) data and digitalization, and (e) administrative and political frameworks. Chapter 5 discusses the implications for policymakers and civil protection organizations based on the answers provided by the interviewees. In the paper’s conclusion, Chapter 6 suggests several aspects where future research could contribute to foster systemic resilience.

## Background: Core Capabilities of Resilient Organizations

Civil protection organizations are governmental, non-profit, or hybrid organizations protecting societies from natural and man-made hazards. Although the public often knows little about how they operate, they fulfill vital tasks during different phases of any crisis to ensure public safety and security (Alexander 2002), most visibly in crisis intervention. Regardless of whether it is an extreme weather event, an industrial accident, or the spread of an infectious disease, civil protection organizations are tasked with minimizing its impact on society, infrastructure, the economy, and the environment while helping society to return to normal.

Civil protection organizations also play a key role in crisis preparedness and long-term adaptation in light of a changing risk landscape. They conduct regular training sessions and scenario exercises, evaluate the handling of previous crises (Lucini 2014; Prior & Roth 2016; Krüger 2019; Berchtold et al. 2020, GRC 2016), and analyze potential future risks using, for example, horizon scanning and other foresight techniques (Roth & Herzog 2016; Geurts et al. 2021; Robinson et al. 2021).

In terms of their capabilities and in view of their many responsibilities, we argue that civil protection organizations are faced with a dual challenge:

First and foremost, they must be able to activate and deploy their core capabilities even under the most trying conditions. The organizations (and the individuals involved) must, therefore, be able to absorb shocking news while maintaining core functions and resuming normal activity as soon as possible. In other words, they must be able to "bounce back" (Darkow 2019). Organizational resilience in the classic sense can be strengthened, for instance by maintaining reliable and robust structures, building up redundancies, and conducting regular stress tests (Boin & van Eeten 2013; Schmidt 2016; REBEKA 2019; Bryce et al. 2020).

Second, and just as importantly, civil protection organizations need the capabilities to deal with emerging challenges for which there are no blueprints or established routines (Stark 2014). To cope with unforeseen crises such as the COVID-19 pandemic, these organizations must be capable of designing and implementing innovation processes (Duchek 2020).

At the same time, dealing with a crisis can lead to comprehensive changes and, as a result, innovation within an organization (see Boukamel et al. 2019, p. 2). This suggests a more dynamic concept of resilience, building on the assumption that complex learning systems are subjected to ongoing change that is necessary to adapt its work processes and maintain performance. Dynamic resilience is strongly dependent on the ability of organizations to quickly respond to specific challenges and shocks by changing their structures and processes (Holling 1973; Walker 2020). This extended view of systemic resilience may be useful for understanding how organizations perform essential functions and services under fluid conditions with many variables, such as a pandemic (Hynes et al. 2020; Roth et al. 2021).

Enhancing the aforementioned capabilities of civil protection organizations is associated with specific conditions and challenges. As these organization belong to the public or non-profit sector, we may assume that they are under much less pressure to be innovative compared to private sector organizations that have to meet short-term customer demands or respond to markets changes (Boin 2019; Rochet et al. 2008; Eckhard et al. 2020). Due to their structures and clearly defined responsibilities, they also appear to be less flexible in their work processes or in implementing innovations (Rainey 2009). However, previous research has also shown that these organizations can be capable of considerable innovation and change, especially under the stress of an immediate crisis (Eckhard et al. 2020; Christensen et al. 2016).

In such extreme situations, innovation often happens through “bricolage” (tinkering)—a term used by Lévi-Strauss (1962) to describe the ability to improvise, solve problems creatively with the resources available, or activate ad hoc networks to create new knowledge based on experiences and the ad hoc solutions that are found. In the context of innovations in public sector organizations, bricolage refers to how innovation happens under conditions of great uncertainty—by deliberately deviating from established protocols or work routines because the established manner of dealing with problems, tasks, or services no longer seems to be working.

Those innovations are usually process innovations. They are created ad hoc by individuals in response to concrete events which makes them barely radical or emerging from a previously given intention. When the initiator of such a bricolage solution discusses it with colleagues and superiors, it is transferred into the organization. In other words, such innovations emerge during its execution, which tends to follow a do-it-yourself principle (Fuglsang 2010).

The question arises as to what can be done to turn bureaucratic organizations into systems fostering improvisation and innovation. Previous research has suggested that the resources organizations can draw on are vital. According to Vollmer (2021), skilled staff as well as a committed management team are particularly important for innovation in disaster management organizations. Analyzing how the SARS pandemic was managed in Singapore in 2003, Teo et al. (2017) identified the importance of tangible resources (finances, technologies, and infrastructure) as well as intangible resources, in particular social capital (which can only be activated in relationships with others), for building organizational resilience.

The experiences of the COVID-19 pandemic and the severe impact they have had on civil protection organizations in many countries has led to a lively debate among scholars and practitioners on the reasons why many organizations were overwhelmed by this event, considering that similar crises had haunted the world before (Sirleaf & Clark 2021; Scharte 2021; Ringsmuth et al. 2022; Center for Security Studies, ETH Zurich 2021). Our study examines this question based on the capabilities that enabled organizations to actively shape learning processes during the COVID-19 crisis and increase their resilience by implementing innovative solutions. Given the timeframe of the pandemic and the enormous demands on civil protection organizations, most of which had no feasible blueprint available to deal with a situation of such dimensions, closer examination should lead to a better understanding of the drivers and barriers of the changes and the dynamics of the resulting innovation.

## Methods

Since few studies were available on organizational learning and innovation during the COVID-19 pandemic, we followed an explorative and thus qualitative approach by drawing on semi-structured interviews with civil protection experts from Austria, Germany, Italy, Sweden, and Switzerland. These countries were chosen based on their particular circumstances at the outbreak of the pandemic in late 2020 and early 2021. Italy was the first European country to be severely affected, forcing it to respond quickly under extreme pressure and uncertainty. Austria, Germany, and Switzerland had to cope with pandemic clusters a little later, giving them more time to prepare. While the three countries have similar civil protection systems, they did not always follow a similar political course during the pandemic and Sweden chose a completely different initial response, at least during the first pandemic wave.

Due to the different impact of the pandemic and the diversity of political and cultural contexts, we chose not to directly compare the civil protection organizations of these countries. Instead, we used a broad empirical basis to explore innovation and learning processes in different settings.

A total of 16 expert interviews were conducted with six representatives of national authorities, five representatives of large aid and relief organizations, and five academic experts whose research focus is on civil protection and security (see Table 1 below).

**Table 1:** Interviewees, countries, and types of organization

Interview No	Role	Organization Type	Country
1	Staff trainer at a large national relief organization	Non-profit	Germany
2	Senior civil protection officer at a large national relief organization	Non-profit	Germany
3	Researcher specialized in risk management, natural hazards, and civil protection	Academia	Germany
4	Freelance consultant, civil protection trainer with a focus on leadership training	Non-profit	Germany
5	Senior researcher specialized in civil protection and crisis management	Academia	Germany
6	Senior official at the federal civil protection authority. Responsible for risk analysis processes	Government	Germany
7	Senior official for national disaster relief at a large national relief organization	Non-profit	Austria
8	Senior official at a national security agency, responsible for civil protection coordination	Government	Austria
9	Senior researcher specialized in civil protection and crisis management	Academia	Austria
10	Senior researcher specialized in preventive medicine, former member of the national Covid task force	Academia	Switzerland
11	Officer of the federal health authority, responsible for innovation processes	Government	Switzerland
12	Senior researcher specialized in societal resilience and civil protection	Academia	Switzerland
13	Senior official at the federal civil protection agency, responsible for risk management	Government	Switzerland
14	Health crisis coordinator and trainer at a large national relief organization	Non-profit	Italy
15	Senior officer at the national firefighting association	Government	Sweden
16	Senior official at the national civil protection agency	Government	Sweden

The authors (i) developed an interview guideline based on existing crisis management and organizational learning theories and a review of official documents and news reports on the management of the COVID-19 pandemic. The structure of the interview guide builds on concepts of dynamic capabilities of organizations in the public and non-profit sector, which are assumed to be embedded in the formal and informal organizational structures (Kattel 2022; Kühl & Schmitz 2013). In order to consider the different requirements of civil protection organizations before, during, and after a critical event, we related these capabilities to the three successive stages of Duchek’s model of resilience (2020).

The interviews were (ii) conducted between June and September 2021 via video conferencing using Microsoft Teams. Each interview lasted between 45 and 120 min. All interviews were recorded, (iii) transcribed, and (iv) coded based on the predefined categories, using the text analysis software MaxQDA. The analysis followed the systematic approach of thematic coding and qualitative content analysis (Flick 2018, p. 473 et seqq.; Mayring 2000; 2014). To achieve a level of intersubjectivity and for quality assurance reasons, the coding was conducted iteratively by various researchers from the pool of authors. As a further step, we (v) selected textual findings, including quotations, for each of Duchek’s three stages (2020).

To validate the interview results, the research team (vi) organized an expert workshop, which was conducted virtually in October 2021. This half-day event brought together 14 representatives of civil protection authorities, aid organization, and research institutions to verify and enhance the main findings of the expert interviews and discuss implications for the future of civil protection in Europe. The aim was to validate our findings, which is crucial to this qualitative approach, and to gain a broader view of the topic, leading to new key issues. Workshop participants received a summary of our validated findings. Subsequently, we (vii) derived consolidated implications for policymakers and civil protection practitioners (see Chapter 5).

## Findings

The findings from the expert interviews illustrate the enormous impact of the COVID-19 pandemic on civil protection organizations. Not only were workflows disrupted and personnel management significantly complicated, but procedures had to be coordinated with partners and ad hoc solutions to unfamiliar problems had to be found. However, in the most acute phase of the crisis, fundamental innovations were hard to achieve. As one interviewee responsible for innovation processes at a governmental agency described the situation:

*“In the beginning, it was always repeated: Crises are opportunities for innovation. But for an organization, it’s actually innovation prevention. You can push certain things forward very quickly by applying pressure, but really renewing yourself, really doing something new, or even disrupting processes have no place during a crisis.” (Interview 11)*

In the initial phase of a crisis, which is characterized by high stress and uncertainty, formal processes and structures are usually more important because they provide some orientation on how to deal with the crisis. Bricolage and, as a result, innovation may be explicitly discouraged at this point. However, we also observed cases of organizations managing to be innovative in order to keep up their core functions as much as possible under the highly dynamic conditions. This happened repeatedly in between the infection waves of the COVID-19 pandemic, but in some cases even while the pandemic was at a peak.

### Workflow and Organizational Structures

Throughout the pandemic, organizations were being challenged to adapt existing work processes to new circumstances and create new organizational structures. To this end, they had to restructure responsibilities and task assignments while the impact of the pandemic was relatively low in order to cope when crisis levels went up:

We [already] used to be flexible, but we [became] more and more flexible so that [during] the starting of each new phase of the pandemic we were already planning for the next phase (Interview 14).

From the perspective of organizational resilience, this result is noteworthy in the sense that capabilities were activated that are usually attributed to coping with the crisis but, on the other hand, already include the anticipation of the next challenge. Reportedly, employees were given a level of individual autonomy and decision-making authority usually reserved for people higher up in the organizational hierarchy. This passing-down of responsibility which appeared to have created new room for independent bricolage and decision-making at lower hierarchical levels was generally welcomed and even seen as a necessity in situations where an organization’s management was overwhelmed by events.

The role of hierarchical structures was viewed ambivalently by the respondents. On the one hand, existing hierarchies which had been tried and tested in previous crises were seen to enable fast and coordinated action. In other instances, however, hierarchies were perceived as obstacles to innovation. One interview partner in charge of innovation processes at a governmental agency described the difficulties during the COVID-19 pandemic as follows:

*Understandably, a very strong top-down structure emerged during the crisis. Innovation is supposed to emerge from below, but nothing could come up from below because the pressure from above was already so strong. (Interview 11)*

One solution reported in this context by a representative of a non-profit organization was to bypass rigid hierarchies using smaller teams with the autonomy to develop and pursue new approaches independently of the "pressure from above" (Interview 14) to ensure more flexibility. This example highlights the precarious relationship between well-established plans and room for flexibility and bricolage, which is also borne out by previous research (Czarniawska 2009, p. 166).

A capability that was found to be particularly important during the crisis was proactive management and deployment of staff members to maintain organizational work processes. This included not only coping with personnel shortages due to sickness, but also responding to the physical and mental health concerns of employees, such as the stress (e.g. due to a lack of social interaction) of working from home.

In some instances, efficient personnel management was able to cope with these demands, e. g. with the help of individual phone consultations. This reportedly worked particularly well when staff members were able to draw on their experiences of dealing with previous national and international crises, such as the influenza pandemic of 2009, or the Ebola pandemic of 2014. Respondents emphasized how important it was to "stay in touch", for example, by talking to employees individually about their well-being.

Activating crisis management teams composed of team members with diverse backgrounds and skillsets was seen as highly effective. Factors such as the professional background, personal commitment, wealth of individual experience, and implicit knowledge of how to handle crisis situations were reported as more important in adapting quickly and identifying ad hoc solutions than formalized work processes and organizational structures.

However, civil protection organizations often struggled to find flexible, creative measures. According to various interviewees, hesitancy to experiment and change familiar work processes was common. The problem with well-established structures was that often, no one felt personally responsible for, or capable of, developing new ideas. The “spirit of openness” (Interview 8) necessary for innovation was particularly difficult to create in security-relevant areas of public administration, where mistakes can have far-reaching consequences.

Symptomatically, one interviewee told us about their attempt to convince superiors at a governmental organization to try out an innovative process to manage multiple tasks during the crisis more effectively:

*And then I went back to the organization and everyone said, ‘Exciting, but […] we don't have a legal mandate, we don't have resources, the timing doesn’t fit'. They were just waiting for new ideas to come from the outside [...], in the end nothing came. (Interview 11)*

Only in a few instances were organizations found to be successful in creating an environment, away from day-to-day firefighting, where innovations could be developed. Our findings indicate that civil protection organizations do not automatically mutate into generators of innovative work processes under crisis conditions but tend to cling to formal structures, making bricolage less likely.

These findings also highlight the importance of effective internal communication and collaborative decision-making as key organizational capabilities. To benefit from the expertise available, often tied to individual employees, it was necessary to create an enabling setting. As a senior disaster relief official working for a large non-profit organization reported:

It takes time for the right people to get in touch with the right players, and that is one of the biggest lessons identified among us." (Interview 7)

In general, internal collaboration appears to have improved primarily when bricolage capabilities came into play, creating new interfaces such as task forces or improving digital solutions. This often requires the combining of the operational expertise of individual employees and making them available in new decision-making situations:

*It is, of course, not the power of the individual but rather a handful of people who regularly throw their experiences into a pot, exchange them among themselves, and reflect on them again and again. (Interview 7)*

In other words, when the expertise of dedicated and creative individuals, otherwise widely dispersed throughout the organization, is brought together and expert knowledge and personal experiences are pooled, bricolage can flourish and take effect.

### Collaboration with Partners and Stakeholders

The interviewees provided interesting insights into how their organizations tried to establish effective and reliable communication channels with external partners and stakeholders during the COVID-19 crisis. Inter-organizational cooperation with regional, European, and global civil protection and relief organizations were seen to add great value to their preparation, response, and adaptation activities. However, it proved extremely difficult to establish such formats of cooperation during the initial stages of the crisis via ad hoc networks; already established and institutionalized networks were more effective (Interview 2).

Cooperation with other types of organizations, for instance in the social or health sectors or in academia, often proved productive as well. Having a wide network of partners and goal-oriented cooperation with different stakeholders were seen as helpful in this regard. Important partners during the COVID-19 pandemic included, among others, psychosocial support agencies, crisis counseling centers, telephone counseling, and general physicians. The cooperation between governmental agencies and technology companies and telecommunication services providers was also found to be productive and a promising foundation for the future.

Our findings are in line with Vollmer’s study on innovation implementation in disaster risk management (2021, p. 140). For example:

*“Commitment of individuals is decisive for successfully implementing an innovation, including commitment at management level, of other staff, as well as external individuals.”* For example, one interviewed expert suggested that such partnerships could be used for innovations in warning systems (Interview 12). Another interviewee working for a governmental agency saw challenge-centered hackathons as a novel approach to take advantage of broad knowledge from tech-savvy partners (Interview 11).

Similar to the findings on internal structures, it became evident from the interviews that external collaborations and networks were also helpful during the COVID-19 crisis, but that newly re-establishing them was extremely difficult. Therefore, organizations mostly tended to use existing networks.

Based on these results, we can now expand our understanding of the bricolage concept in a crisis management situation. Their networks enable those ready to engage in bricolage to collaborate with others and draw on the advice of well-known external specialists rather than just relying on their own experiences and assessment of a given situation. This makes bricolage a network-based or collaborative crisis coping mechanism, as is further illustrated in the next paragraph.

While involving a diversity of players appears to have facilitated the management of the COVID-19 pandemic, we also found that it can make collaborative work more demanding. A senior executive at a relief organization described some of the conflicts that occurred in a collaborative crisis unit he participated in during the pandemic:

*And the differences in the unit were then at some point so clear and, above all, the pressure on our own organization so great that we virtually divided [the unit], on the basis of content and personnel. (Interview 7)*

Nevertheless, the exchange with external partners is important, especially in terms of recognizing new challenges at an early stage and receiving impulses for innovative approaches or solutions. In situations where an international exchange of knowledge had been established, adaptation to new situations was reported to happen more smoothly. The joint development of key definitions and concepts proved to be effective in improving the cooperation between different partners. Since important key terms such as critical infrastructure, resilience, and crisis preparedness had often not been clearly defined, developing a unified terminology served as a basic prerequisite for structured and joint action:

First, we have to find a common language. We first have to develop the same understanding of different methods. (Interview 7)

If a basis for cooperation already existed before the pandemic, it was easier to find this “common language” and save valuable time. Collaborations with other organizations are especially important in the anticipation phase to better foresee potential challenges as well as in the coping phase to more easily coordinate appropriate actions. The results therefore show that a bricolage strategy in a crisis situation cannot be created on a greenfield basis but needs to rely on existing structures, existing networks, and a common language.

### Crisis Preparedness and Risk Analysis

Risk analyses are seen as useful tools that enable organizations to adapt more quickly and effectively to new challenges and trends and to expand their range of capabilities in crisis preparation. In the context of the COVID-19 pandemic, underdeveloped risk analysis capabilities appear to have been a main contributing factor to insufficient levels of preparedness. A prominent example is the lack of personal protective equipment at the beginning of the pandemic, as reported by an interviewee working for a governmental organization:

*And the whole country Sweden was not prepared, because in Sweden for many years now, we have thought that the Russians are not the bad guys anymore so we don’t need to have any military, we don’t have to have any warehouse full of protection materials or things like that. So, Sweden was really bad out when the pandemic started. In a lot of places, like health care service, […] they were working without any protection because there was none. It was empty. [...] We were not prepared really. (Interview 15)*

In general, the COVID-19 crisis demonstrated several limitations of current approaches to risk analysis. Some interviewees reported a need for better foresight processes, horizon scanning methods, and increased consideration of continuously updated scenarios. In addition, a crisis management budget and more, and better trained, risk analysis experts could have helped facilitate this process. Similarly, closer ties and better communication with critical infrastructure operators would have been useful. In addition, some interviewees called for a stronger focus on vulnerabilities to understand what “brings an organization down” in a crisis (Interview 6). Above all, however, it was reported that risk analysis had frequently not been integrated into the organization’s preparedness planning at all.

An important reason for the lack of planning and inadequate risk analysis was felt to be the low levels of political attention to disaster preparedness. As a senior disaster management specialist put it bluntly, disasters are “not sexy” and, because of that, interest will rapidly decline after an acute disaster or crisis (Interview 9). As a consequence, scientific risk analyses were often not taken seriously enough in the past. It was remarked that scientists rarely receive the necessary attention at the political level. This may be partly due to the fact that the relevant research studies have too few concrete implications and recommendations aimed at politicians. Accordingly, it was seen as a key capability of organizations to generate attention for crisis preparation and develop recommendations for action.

### Data and Digitalization

All organizations were reported to be seeking ways to advance the digitalization of their structures and processes. The increased aggregation, processing, and use of large data sets for a variety of purposes has been a topic of concern for many organizations, even before the pandemic. One prime use case is the application of big data to enhance situational awareness of decision-makers. By 2019, however, few if any civil protection organizations were ready for a bold move toward digitalization. Instead, according to some interviewees, they continued to operate more or less as before. One of the reasons cited was that the benefits of digitalization were often not directly visible in the short term.

This finding is surprising, given that the COVID-19 crisis revealed numerous possible applications for digital innovation. For example, in the absence of a digital register of deployed and available response personnel, civil protection organizations had to improvise and find spontaneous (technical) solutions. One researcher interviewed described how data from the private sector might have been useful but has, so far, remained largely unused:

*But of course, there's actually already a lot of data floating around or that we have, especially in terms of the smartphone, and it's sitting either with the big tech companies or with telecoms. (Interview 12)*

The heterogeneity of data structures further complicates the harmonization of situation analysis. This often involves definitional questions, such as "what exactly do we mean by an intensive care bed?" While interviewees frequently complained about a lack of software or outdated applications, they also pointed to the importance of sufficient IT capabilities within the organization and the need for innovative software to use during a crisis. An important barrier in this context appears to have been a rather common hesitancy at the management level when it came to technological innovations. Asked about hindrances, one researcher from Germany complained:

*Our country has never been an early adopter but takes its time. And the question is, if you introduce a new technology, let's look at artificial intelligence or drones, (...) you have a much greater risk averseness. (Interview 3)*

Accordingly, there is great, untapped potential for innovation resulting from the deployment of innovative technologies around the use of artificial intelligence and cutting-edge sensor technology. These technologies encompass, for example, digital twins for situation assessments, AI analytics of big data sets including social media sentiment analysis, image recognition for hazardous material labels, situation development analysis, AI-based leadership training, and algorithms for predicting peak workloads for first responders (Illing 2020).

Since digital capabilities appear to be underdeveloped in civil protection organizations, it often proved hard to identify clear use cases in the context of the COVID-19 crisis. One senior official at a large national relief organization described how the "vast amounts" of data generated during the pandemic would now have to be used in a way to understand the organization itself. He concluded:

*One of the big challenges I see right now is to systematically process all the knowledge that we have gained in the organization in one way or another to actually gain a benefit from it. Knowledge management and evaluation capacities are therefore seen as important elements in order not to lose an organization’s know-how. Otherwise, there might be the risk that lessons disappear in a drawer. (Interview 7)*

State-of-the-art means, such as AI, could be used to evaluate data, both quantitatively and qualitatively, and simplify data exchange (Interviews 4 and 7).

However, the structures needed to do this are mostly still being developed or not yet in widespread use. In this context, data protection was a concern for many of the organizations interviewed. Accordingly, the know-how needed for efficient data collection and evaluation that complies with data protection requirements is becoming increasingly important. In some cases, peer structures and learning mentorships were established over the course of the pandemic to support the less digitally proficient volunteers working for a national relief organization (Interview 1).

Such structures can help to better embed digitalization within civil protection organizations in general.

### Administrative and Political Framework

The administrative and political frameworks of the countries studied are quite different and therefore demand diverse capabilities from the respective organizations. On the one hand, the decentralized structures of civil protection in Austria, Germany, and Switzerland allowed the local civil protection organizations to be flexible in adapting measures to context-specific conditions. On the other hand, without a centralized system, data collection and dissemination as well as logistics and financing were more difficult and sometimes redundant, and the implementation of measures was difficult to control. This required organizations to involve strategic or political levels in their processes. Where these kinds of collaboration already existed, administrative hurdles were perceived to be lower and organizations were given greater room to maneuver (Interview 4).

Some organizations had to partner with a number of stakeholders to balance the desire for uniform solutions with decentralized decision autonomy. To this end, organizations had to employ cooperative capabilities. They had to become more visible, and they had to bring different disciplines to the table without loss of focus. Asked about the employability of concepts used in previous crises, one representative of a large national relief organization stated the following:

*In a democratic system, there are simply too many stakeholders who, for a variety of reasons, also have a say in the matter. And then, of course, the best concept can dissolve again in the infinity of discussion. (Interview 7)*

Some respondents also complained that representatives at the political level had often had an insufficient grasp of the situation and had been overwhelmed by the dynamics of the pandemic, leading to short-sighted and ineffective operational decisions at odds with civil protection expertise. Accordingly, civil protection organizations needed to develop the ability to provide targeted information to policymakers. A senior scientist, a high-profile member of a national Covid task force, argued that civil protection experts should "really impose the information onto them (the policymakers)" (Interview 10).

In this context, a change in self-perception appears to be emerging among civil protection organizations. This helps them make the transition from executing entities to embracing the roles of service provider, information broker, and decision support agent for policymakers.

## Implications for Stakeholders

The insights gained by studying civil protection organizations and their work during the COVID-19 pandemic provide a number of relevant implications that can help improve systemic resilience in the face of potential future pandemics and catastrophic events. To validate the results, our findings were discussed in a stakeholder workshop with representatives from academia, non-profit, and governmental organizations. These discussions resulted in the following lessons learned aimed at policymakers and civil protection organizations.

### Lessons for Policymakers

Although a conclusive assessment is not possible at present, the COVID-19 pandemic has clearly shown that systemic resilience needs to be strengthened to be able to respond and adapt to large-scale, dynamic crises in a more coordinated and effective manner. While the COVID-19 crisis was at its peak, there was hardly any room for reflection for those involved. Once the acute crisis stage has been addressed, however, policymakers should focus on improving resilience and allocating resources. They should also adopt mechanisms to support this process.

However, comprehensive assessment and evaluation processes must not result in some form of "analysis paralysis". Overthinking and overanalyzing possible measures prevents actual decisions from being made and measures from being taken. It may be expected that, if reforms are delayed for too long, the political will for fundamental structural change dwindles. A crisis like the COVID-19 pandemic offers a “window of opportunity” for change and transition (Birkmann et al. 2010; Bodenheimer & Leidenberger 2020). Thus, the plea based on the findings is to utilize political momentum as “windows of opportunity” (Birkmann et al. 2010).

One area where the pandemic has revealed an urgent need for political action is the creation of appropriate legal conditions for a rapid and comprehensive exchange of crisis-related data. Facilitating the sharing of information among authorities at different administrative levels and with operators of critical infrastructures seems to be a prerequisite for increasing the capabilities of civil protection organizations in a hyper-linked and increasingly digitalized world, for example, to conduct complex, integrated risk analyses and increase situational awareness during a crisis (see Lauwe et al. 2019).

One way to foster innovation can be to build up data infrastructure settings that enable the sharing of existing municipal infrastructure data also for civil protection purposes and serve as a common basis for various purpose-oriented digital solutions (Sautter et al. 2021a), such as a cross-organization tool for intensive care bed management (Result of the Expert Workshop).

Furthermore, policymakers should establish the necessary institutional framework conditions for regional and international coordination and cooperation (Results of the Expert Workshop). The results from the survey show that a reliable and trusted network with international partners is an important source of resilience. In particular, policymakers need to strengthen the European Civil Protection Mechanism (ECPM) as the central framework for coordination and solidarity on the continent and better align national policies with the ECPM.

Finally, we also feel that political action is necessary to change the defensive risk culture that seems to prevail in civil protection organizations (Results of the Expert Workshop). Underdeveloped error tolerance and general risk averseness make it difficult for organizations to try out novel approaches. Here, policymakers should create the framework conditions for experimental learning processes and provide incentives for genuine innovation.

Politicians alone have the mandate to initiate this kind of systemic reform. In most European countries, the existing structures and responsibilities in civil protection date back to the Cold War era. The subnational level, which is primarily oriented toward local and comparatively frequent events (e.g. regional floods, forest fires, and earthquakes), plays a prominent role in these systems. As a result, information, skills, and competencies tend to be widely dispersed over a broad range of players. Reforms of national civil protection authorities, such as the ongoing restructuring process of the German Federal Office of Civil Protection and Disaster Assistance (BBK), could be used to better connect existing resources and build up new capacities when needed (Center for Security Studies, ETH Zurich, 2021; Voss 2021, p. 23).

### Lessons for Civil Protection Organizations

For civil protection organizations to better prepare for future risks and disasters, they must have the ability to critically and openly reflect on the lessons from the COVID-19 crisis. To this end, several organizations have already initiated self-evaluation processes, which were consistently perceived as beneficial by the interviewees. In some cases, however, the involvement of external experts appears to be indicated. International networks can also make valuable contributions to ensure the exchange of experiences and a more permanent adaptation of lessons learned and good practices.

The reflection and learning process should consider how internal processes and structures can be overhauled so that civil protection organizations can respond more quickly and more appropriately to future crises. Although action plans for various crisis scenarios had been in place, the scale of the COVID-19 pandemic with its all-encompassing societal implications required novel approaches and an expansion of existing capabilities. Such concepts, including volunteer engagement, have been described in the literature (Zettl et al. 2018; Zettl 2018). However, there is still a lack of implementation experience. Under much pressure to act while adapting to the novelty of the crisis, civil protection organizations were virtually forced to be pragmatic, flexible, and collaborative. They were found to be most effective where rigid hierarchies were overcome by enabling small and diverse teams to self-organize and develop innovative solutions.

Less hierarchy also means allowing operational staff take on more responsibility. During the COVID-19 pandemic, employees on the ground were forced to make far-reaching decisions and were trusted by their superiors because they had the specific expertise needed.

As one of the lessons from our research, we recommend that civil protection organizations should establish permanent organizational principles and frameworks to strengthen self-responsibility and -organization, for example by complementing traditional organizational structures with bricolage structures for fostering improvisation and increase organizational resilience (Weick, 1993). Based on our findings, such bricolage structures could include, for example, established relations with external specialists, temporary working groups (task forces), ad hoc groups of creative and particularly conscientious employees, or access to structured big data on how one's own organization or others had dealt with a crisis (Watson et al. 2017). Such structures can provide those involved in bricolage with additional building blocks to develop ad hoc solutions and create innovations.

After the pandemic, it will be vital to evaluate which of the processes or structures created out of necessity should be kept, and which processes should go back to ‘normal’. A great deal of institutional knowledge was generated that can also be helpful in future crisis situations. An important task in the coming period will therefore be to scale back structures that are no longer needed without permanently losing the skills and knowledge that have been acquired.

Approaches should be developed to re-activate these resources quickly when needed and adapt them to new challenges. A prerequisite for this is the creation of institutionalized knowledge management structures to supplement underlying data management structures. Central data infrastructures following the FAIR criteria (findable, accessible, interoperable and reusable) could provide valuable information, previously unavailable due to an outdated silo mentality (Sautter et al. 2021a).

In addition, it is desirable to create structures that enable scientific expertise to be incorporated more effectively into crisis management. Important criteria here are ensuring the independence of researchers and the inclusion of different scientific disciplines.

Civil protection organizations should invest more in close ties with their partners and stakeholders. In the event of a crisis, inter-organizational and international cooperation partners can be important sources of impetus and should therefore be established in advance. At the beginning of a cooperation, it is important to establish common definitions and methods in order to find a common language and a common interpretation of the crisis requirements, especially in interdisciplinary exchange. According to knowledge and data management experts, this should also be reflected by corresponding metadata, taxonomies, and ontologies (Pottebaum et al. 2016; Sautter et al. 2021a).

Good contacts to non-governmental organizations, associations, and civil society in general can help in a crisis to identify where help is needed in order to coordinate and activate important resources. Low-threshold offers, such as platforms for volunteers (Neubauer et al. 2013) are helpful for involving citizens in an effective and safe way. Further, it seems important to institutionalize partnerships with civil society groups. The aim here should be to involve them already in crisis prevention, especially representatives of vulnerable groups (Zettl et al. 2018). To encourage the participation of civil society in civil protection-related topics, public communication is a key capacity of any civil protection organization that needs to be strengthened (Eckard et al., 2020).

Finally, our findings highlight the need to accelerate investment in digital capacities. In the course of the pandemic, digitalization affected practically all areas of work in the organizations studied, from internal communications and resource planning to situation assessment. The rapidly growing amount of data of various kinds has many applications, including enhanced situation assessment and resource planning. At the same time, the flood of data available also poses new challenges, both in terms of the technology used and for the users. In order to continue the renewal of processes that have been initiated, it is important to invest more in the digital skills of employees. In recruitment, digital skills should already be considered a core capability for junior staff, and they are becoming increasingly important in more and more areas. In this context, it must be considered that not all employees have the same prerequisites for dealing with digital technologies. Learning mentorships within organizations can provide support and promote a sense of community among employees.

According to Nestler (2014), it is also important to emphasize that human-technology interaction for civil protection practitioners is not only a big challenge in crisis situations (coping) but also in anticipation and adaptation phases. In fact, it can potentially decide over life and death. At the same time, he found that the efficiency and usability of digital tools depends very much on their user-oriented design.

Finally, decision-makers in civil protection organizations should be sensitive to the usability of their technical tools, especially in procurement processes but also during the operation of systems (Sautter et al. 2021b).

## Conclusions

Using the example of the COVID-19 crisis, our study provides insights into the mechanisms enabling civil protection organizations to learn, be innovative, and improve their capabilities during a crisis to build greater organizational resilience. In our study, we focused on organizational conditions enabling civil protection organizations to actively shape crisis-related learning processes, particularly in the areas of workflow and organizational structures, collaboration with partners and stakeholders, preparedness and risk analysis, digitalization, and the political situation. Due to the explorative character of the study which is comparing a number of different organizations operating in different settings, we cannot provide direct comparisons or directly conclude which organizational capacities are necessary for a successful pandemic management. We were, however, able identify the factors, skills, and tools that allowed organizations to adapt to the pandemic effectively.

To adapt their work processes and organizational structures during the COVID-19 pandemic, civil protection organizations had to act quickly and be flexible. This enabled them to spontaneously reallocate responsibilities and tasks and to produce and adapt creative solutions where infrastructure was lacking. In this process, organizations were capable to develop innovations and build new capabilities that are likely to remain in operation in readiness for future pandemics and other disasters. At the same time, organizations were able to build on existing capabilities and experience from previous crises.

In order to benefit from collaborations, new task forces had to be established ad hoc and creative solutions had to be found to overcome innovation obstacles. Here, it became clear that the development of new capabilities and innovations is often difficult in the acute phase of crisis management. In contrast, already established communication channels and cooperation were beneficial for collaboration during the crisis (see also Voss 2021, p. 19). The sometimes widely varying administrative and political framework conditions required the organizations to explore any space to maneuver flexibly and creatively. As pointed out by the Center for Security Studies at ETH Zurich (2021), cooperation was often hampered by distributed state- and federal-level responsibilities and insufficiently prepared authorities.

The notable increase in the need for digital networks, data collection, and data sharing during the pandemic highlighted a deficiency in data management and digitalization, but also prompted some organizations to implement changes in order to quickly process data during the crisis and analyze it in a targeted manner during the adaptation phase. Bricolage skills, such as spontaneously establishing learning mentorships and ad hoc data sharing networks, were particularly helpful. Other organizations were slower to build these capabilities, often still relying on previous skills and solutions.

Identifying important lessons and drawing the right conclusions for the future will be an important task for policymakers and civil protection organizations in the months and years to come. However, this process can be supported by the scientific community, for example by developing practice-oriented definitions of key concepts or by supporting practitioners with scenarios, trend analysis, and horizon scanning (Gerhold et al. 2019; Neisser & Kox 2021).

The necessary self-evaluation steps toward organizational learning, which must primarily be steered from within the organizations, can also be supported by scientific or consultancy analyses. Applied research could benefit from working more closely with digital companies to develop innovative solutions for interagency data infrastructures. These solutions should be developed specifically for inter-organizational crisis management and should include common terminologies, and data structures, usability, and flexibility. The exchange of data sets between scientists and civil protection practitioners via joint initiatives can be of immense help here.

At the time of writing, the COVID-19 crisis was still ongoing. Consequently, organizations were still adapting to the changed setting. In this context, it is essential to point out how differently the organizations had to shape their response to a perpetuated, global pandemic. Civil protection organizations traditionally work along the crisis cycle: After a shock or disaster event, there is a crisis response period followed by a defined recovery phase, which ends with the return to normal. The COVID-19 pandemic, however, hit countries in repeated and sometimes overlapping waves of varying intensity. Therefore, organizations had to master the challenges of recovering from a previous wave while responding to the ongoing crisis and while preparing for the next wave. This complex and combined pressure on the organizations marks a challenge of a new quality and magnitude. Flexibility and adaptable processes for inter-crisis learning will become an essential quality for civil protection and aid organizations to pursue in order to achieve organizational resilience.

## Supplementary Information

Below is the link to the electronic supplementary material.

Supplementary file1 (DOCX 117 kb)

Supplementary file2 (XLSX 27 kb)

Supplementary file3 (DOCX 25 kb)

## Data Availability

The metadata of the datasets generated and analyzed during the current study will be made available to the public in due course. Due to data protection and the purpose orientation of the data used, full data is available upon request for Fraunhofer-researchers only (please provide details of the research purpose and affiliation for this). Roth, Florian; Kaluza, Benjamin; Pfeffer, Katharina; Rümelin, Esther; Kirchner; Joel; Overmeyer; Maike, Neisser, Florian; Jackwerth-Rice, Thomas; Kilicaslan, Aleyna; Sautter, Johannes: Innovation in Times of Crisis: How Civil Protection Organizations in Europe Coped and Adapted During the COVID-19 Pandemic: Semi-structured interviews with civil protection experts from Austria, Germany, Italy, Sweden, and Switzerland; Dataset, Fraunhofer-Gesellschaft, 2023, http://dx.doi.org/10.24406/fordatis/249.
