# Supplementary material for: Innovation in Times of Crisis: How Civil Protection Organizations in Europe Coped and Adapted During the COVID-19 Pandemic
Source: Eur J Secur Res. 2023 May 9:1–23. Online ahead of print. doi: 10.1007/s41125-023-00090-6 (PMC10169097; doi:10.1007/s41125-023-00090-6)
Supplement: Supplementary file 1 — Supplementary file1 (DOCX 117 kb) [file 41125_2023_90_MOESM1_ESM.docx]

**KResCo - Interview Guideline**

Interview guide (Online Resource) for **European Journal for Security Research** article submission: **Innovation in times of crisis: How European civil protection organizations coped and adapted during the COVID-19 pandemic**

by

Florian Roth, Fraunhofer Institute for Systems and Innovation Research (ISI), Karlsruhe, Germany, florian.roth@isi.fraunhofer.de, ORCID 0000-0002-5287-6448
Johannes Sautter, Fraunhofer Institute for Industrial Engineering (IAO), Stuttgart, Germany
Benjamin Kaluza, Fraunhofer Institute for Technological Trend Analysis (INT), Euskirchen, Germany Katharina Pfeffer, Fraunhofer Institute for Systems and Innovation Research (ISI), Karlsruhe, Germany
Esther Rümelin, Fraunhofer Institute for Systems and Innovation Research (ISI), Karlsruhe, Germany Joel Kirchner, Fraunhofer Institute for Industrial Engineering (IAO), Stuttgart, Germany
Maike Overmeyer, Fraunhofer Institute for Technological Trend Analysis (INT), Euskirchen, Germany Florian Neisser, Fraunhofer Institute for Technological Trend Analysis (INT), Euskirchen, Germany Thomas Jackwerth-Rice, Fraunhofer Institute for Systems and Innovation Research (ISI), Karlsruhe, Germany
Aleyna Kilicaslan, Fraunhofer Institute for Industrial Engineering (IAO), Germany

# 1) Introduction

Introduce objectives and overall research question of the project

*1. Please briefly explain what your role and responsibilities are in the organisation.*

*2. What were your responsibilities during the Corona crisis?*

*3. How did your organisation experience the crisis last year?*

Agree with interviewee on the focus of the discussion (suggestion: first wave of infection)

# 2) Crisis prevention ("anticipation" before the first wave of infection)

| **Topic** | **Question** | **Secondary Questions** | **Detailed aspects** |
| --- | --- | --- | --- |
| Biggest challenges in this phase | *1) Could you describe at the beginning to what extent you were already prepared for the Corona crisis?* | What were the biggest challenges for your organisation in assessing the risks of the Corona crisis in advance? |  |
|  | *2) To what extent did your organisation try to assess the risks of the pandemic in the run-up to the crisis? Could you give an example?* | Which roles, persons or departments worked particularly closely together in this phase?  What role did cooperation with partners play here (at home / abroad, especially in Europe)?  How do they benefit from the national risk analysis? | - Crisis / emergency plans - Improvisation capability - Scenario & risk analyses - Safety management system |
| Capabilities and handling of data | *3) What capabilities first enable your organisation to adequately assess the risks of such a crisis?* | Which of these capabilities were already available internally before the crisis?  To what extent were these capabilities only activated in interaction with external parties (domestic / foreign)? (Authorities, science, etc.)  Could you describe this with an example? | - Focus on cooperation with other European countries / European cooperation |
|  | *4) What data was particularly important for your organisation to adequately assess the risks?* | From which internal or external sources do you obtain this data?  For what purposes was this data used?  What data was missing?  What did you learn about data use and management during the crisis? |  |
| Innovation | *5) To what extent were working procedures or organisational structures changed during this phase in order to better assess the risks?*  *6) To what extent were new digital solutions introduced to better assess the risks?* |  |  |

# 3) Acute crisis management ("coping" during the first wave of infection).

| **Topic** | **Question** | **Secondary Questions** | **Detailed aspects** |
| --- | --- | --- | --- |
| Biggest challenges in this phase | *1) Could you describe what measures your organisation took when the crisis hit, and infections increased?* | What were the biggest challenges for your organisation in this phase?  What data was particularly important in this phase?  From which internal or external sources did you obtain this data?  For what purposes was this data used?  What data was missing to better manage the crisis? |  |
|  | *2) How has the organisation proceeded to take / develop appropriate measures for upcoming challenges?* | Which roles, persons or departments worked particularly closely together in this phase?  What role did cooperation with partners play (at home / abroad, especially in Europe)? E.g., with public authorities, science | To what extent did the EU-coordinated pan-European cooperation play a role?    Have you received or provided assistance beyond that? |
| Capabilities and handling of data | *3) What capabilities enable your organisation to develop / take appropriate measures to deal with the crisis?*  *4) To what extent were these capabilities only activated in cooperation with external parties (at home / abroad)?* | Which of these capabilities were already available internally before the crisis?  To what extent were these capabilities only activated in cooperation with external parties (at home / abroad)? (Authorities, science, etc.)  Could you give an example? | - Implementation of emergency plans - Technical redundancies - knowledge management, documented experience, networks - - Ability to improvise - trustworthy information / cooperation (internal, external) - leadership behaviour - Evidence-based DRM |
|  | *5) How and regarding which topics did your organisation communicate with the public?* |  | - Strategy - Channels of communication |
|  | *6) What data was particularly important for your organisation to adequately assess the crisis?* | From which internal or external sources do you obtain this data?  For what purposes was this data used?  Which data was missing?  What did you learn about data use and management during the crisis? |  |
| Innovation | *7) To what extent were working procedures or organisational structures changed during this phase in order to better assess the risks?*  *8) To what extent were new digital solutions introduced to better assess the risks?* |  |  |

# 4) Crisis aftermath ("adaptation" after the first wave of infection)

| **Topic** | **Questions** | **Secondary Questions** | **Detailed aspects** |
| --- | --- | --- | --- |
| Biggest challenges in this phase | *1) Could you describe how your organisation has specifically evaluated its own experiences with the crisis in order to be better prepared for future crises?* | What were the key findings? | To what extent has the learning been retained?  How does your organisation pass on the new knowledge to a new generation of staff? |
| Capabilities and handling of data | *2) What data was particularly helpful at this stage?* | From which internal or external sources do you obtain this data?  Which data was missing?  What did you learn about data use and management during the crisis? |  |

# 5) Expectations regarding policy making

# *1. What would you recommend to German or European policy makers to improve crisis management?*

# *2. What can policy maker do concretely to strengthen the contribution of its organisation to crisis management?*

# 6) Closing remarks

*1. is there anything else you would like to say at this point?*

*2. Can you think of anyone else with whom we could also talk about this topic again?*

*3. If you have any questions or comments afterwards, please do not hesitate to contact us.*

Thank you very much for this interview.
