# Supplementary material for: Innovation in Times of Crisis: How Civil Protection Organizations in Europe Coped and Adapted During the COVID-19 Pandemic
Source: Eur J Secur Res. 2023 May 9:1–23. Online ahead of print. doi: 10.1007/s41125-023-00090-6 (PMC10169097; doi:10.1007/s41125-023-00090-6)
Supplement: Supplementary file 3 — Supplementary file3 (DOCX 25 kb) [file 41125_2023_90_MOESM3_ESM.docx]

## Appendix III

Table 1. Expert workshop participants (Online-Meeting in October 2021)

| **Participant No.** | **Role** | **Organisation Type** | **Country** |
| --- | --- | --- | --- |
| 1 | Commander with overall responsibility in a civil protection organization | Nonprofit | Austria |
| 2 | Member of a crisis team | Nonprofit | Austria |
| 3 | Personnel development/further training for management and teaching staff regarding aid and relief | Nonprofit | Germany |
| 4 | Civil Protection Officer | Nonprofit | Germany |
| 5 | Freelance consultant, trainer in the field of civil protection with a focus on leadership training | Nonprofit | Germany |
| 6 | Manager in a large plant fire department | Private | Germany |
| 7 | Pedagogue in a training center for civil protection, disaster preparedness and internal and external security | Private | Germany |
| 8 | Responsible for personnel development, further training and education | Nonprofit | Germany |
| 9 | Professor of anglistic and cultural studies | Academia | Germany |
| 10 | Chairman of a civil protection interest group |  | Germany |
| 11 | Reserach associate for media and communication sciences | Academia | Germany |
| 12 | Reserach associate at a institute für media research | Academia | Germany |
| 13 | Division Head of a federal agency | Governmental | Switzerland |
